# Two constructed wetlands within a Mediterranean natural park immersed in an agrolandscape reduce most heavy metal water concentrations and dampen the majority of pesticide presence

**DOI:** 10.1007/s11356-022-21365-w

**Published:** 2022-06-17

**Authors:** Maria A. Rodrigo, Eric Puche, Nuria Carabal, Sergio Armenta, Francesc A. Esteve-Turrillas, Javier Jiménez, Fernando Juan

**Affiliations:** 1 Integrative Ecology Group, Cavanilles Institute of Biodiversity and Evolutionary Biology, University of València, Catedrático José Beltrán 2, 46980 Paterna, Spain; 2 Department of Analytical Chemistry, University of Valencia, Dr Moliner 50, 46100 Burjassot, Spain; 3 Hidraqua Gestión Integral de Aguas de Levante, S.A. Carrer de Sant Sebastià, 12 Alfafar, 46910 Valencia, Spain; 4 Aguas de Las Cuencas Mediterráneas, S.A. (ACUAMED), Pasaje Doctor Serra 2, 3º planta, 46004 Valencia, Spain

**Keywords:** Domestic wastewater, Tertiary treatment, Metals/Metalloids, Herbicides, Fungicides, Insecticides, Rice agriculture

## Abstract

The water concentrations of 12 heavy and other metals/metalloids were analyzed seasonally along two horizontal-flow constructed wetlands (CWs) (Tancat Mília—TM and Tancat l’Illa—TLI) located within the Mediterranean *Albufera de València* Natural Park during 2020–2021. A wide-scope screening of pesticides present in waters was also performed. The two CWs were created to improve water quality and increase biodiversity. They currently receive effluent waters from two different tertiary-treatment wastewater plants, and the water flows along the CWs before being discharged into the main lagoon and a smaller lagoon in TM and TLI, respectively. TLI manages to reduce (Mn) or maintain the concentration of most of the studied elements (Zn, Ni, Hg, Cr, Fe Cd, Cu) at the same level as outside (67%). Only Al, Pb, B, and As remain at a higher concentration. TM also reduces Zn and Cu and keeps the concentration of Cr, Cd, and Hg (representing 42%). Al, Pb, B, and As remain at higher concentrations, as in TLI, but Ni, Fe, and Mn are also at higher concentrations. Although both CWs vary in their ability to remove elements, no risks to human health or the environment have been detected due to the low metal concentration in their outlets, all of them (except Hg) below the legal limits for environmental quality in the European Union. With the detection of 71 compounds in water in each CW area (26 herbicides, 26 insecticides, and 19 fungicides in TLI, and 29 herbicides, 23 insecticides, and 19 fungicides in TM), we also provide evidence of the impact of pesticides, which depends on the application method (helicopter, tractor), originated from areas with high agricultural pressure (chiefly rice crops) on systems (mainly TM) created to preserve biodiversity. Nevertheless, both systems provide crucial environmental services in water quality in this agrolandscape.

**Supplementary Information:**

The online version contains supplementary material available at 10.1007/s11356-022-21365-w.

## Introduction

Constructed wetlands (CWs) as ecological and alternative solutions for wastewater treatment have proven effective worldwide, particularly for nutrient reduction (Kadlec and Wallace 2008). However, the studies on CWs being used to reduce other kinds of pollution, such as priority hazardous substances, from municipal wastewater treatment plants before being discharged into natural and protected areas are scarce (Arroyo et al. 2010; Pedescoll et al. 2015). Wastewater, even treated, may contain undesirable chemical constituents such as heavy metals and pesticides, particularly in highly populated areas with intensive agricultural activities, such as coastal areas in the Mediterranean. The presence of both kinds of substances can be related, since, for example, fertilizers are usually not sufficiently purified during the manufacturing processes, for economic reasons, and usually contain several impurities, including heavy metals. Moreover, heavy metals often form a part of the active compounds of pesticides (Gimeno-García et al. 1996). Many other human activities introduce metals into the environment (e.g., mining, traffic, industries, wastewater treatment plants, waste landfills, etc.) (Hernández-Crespo and Martín, 2015). Moreover, tertiary treatments in wastewater treatment plants using chemical reagents (coagulants, flocculants, etc.), to increase the removal efficiency of phosphorus and other nutrients (Iborra-Clar et al. 2011), may represent a source of certain elements being discharged into sensitive ecosystems such as wetlands within natural parks.

The global term “heavy metals” sometimes includes other elements belonging to the periodical system, which are in fact not metals (e.g., boron), nor are they “heavy” (e.g., aluminum, manganese, iron), but they can become a serious environmental problem (e.g., acute and chronic toxicity to aquatic organisms, accumulation in the ecosystem, and losses of habitats and biodiversity; Directive 2006/11/EC; Yu et al. 2021). Within the term pesticide, a broader spectrum of substances is included, such as herbicides, insecticides, fungicides, but also repellents, growth regulators, etc. (European Commission 2020). Pesticides are considered as pollutants by the Water Framework Directive (WFD, Directive 2000/60/EEC), and are considered priority substances by Directive 2013/39/EU, being categorized as dangerous substances by Directive 2006/11/EC. In fact, monitoring studies have reported the presence of a large number of these types of contaminants in water bodies across Europe (Houtman 2010; Hedge et al. 2014; Rico et al. 2016, 2019). Furthermore, this chemical pollution is highly related and affects some other water quality parameters, depicting a complex amalgam of factors which have to be taken into consideration (Rico et al. 2016).

The *Albufera de València* Natural Park is one of the most important wetlands in the Iberian Peninsula and the Mediterranean zone: it has been protected by the Ramsar Convention on Wetlands since 1990; mentioned as a special protection area (SPA) by the Birds Directive (Directive 2009/147/EC) since 1991; included in the Natura 2000 network (MITECO 2021) as well as being classified as a Site of Community Importance (SCI) by the Habitat Directive (Council Directive 92/43/EEC) in 2006. However, currently, the area is still subjected to degradation, which started at the beginning of the 1970s (Dafauce 1975). Intensive agriculture, urbanization, and industrial activities are the main causes of said deterioration (Soria 2006). For example, metals such as iron, zinc, and copper have been applied to rice-growing soils in the form of pesticides at levels of 1–15 kg/ha per year, whereas cadmium has been applied in fertilizers at amounts of 150–450 mg/ha per year (Boluda et al. 1993).

Among the several initiatives implemented within the *Albufera de València* Natural Park to reduce water pollution, one of them is the construction of the above mentioned CWs surrounding the main lagoon (called the *Albufera de València*) (Martín et al. 2013; Rodrigo et al. 2013). These CWs were created from the transformation of former rice fields. These artificial wetlands were also constructed to recover lost habitats in the natural park they belong to, so as to improve biodiversity both inside and outside the CWs (Hernández-Crespo et al. 2017; Rodrigo and Segura 2020). Two of these CWs are located south of the main lagoon, and currently are exclusively fed by the effluents of two wastewater treatment plants. Moreover, the CWs are immersed in a landscape devoted mainly to rice agriculture, where pesticide treatments were and continue to be applied (Calvo et al. 2021).

Several studies of heavy metals and pesticides in the aquatic habitats of the *Albufera de València* Natural Park have been carried out (see Gimeno-García et al. 1996 for the first and Calvo et al. 2021, and references therein, for the latter). However, the performance of the CWs within this Natural Park in relation to heavy metals and pesticides has never been approached. Thus, the aim of this study is to assess how the wastewater effluents, as water supply to the CWs, impact on the changes in the concentrations of said elements and compounds as water passes through these systems. We hypothesize that most elements/compound concentrations in the inlets will be reduced, while others may be increased and this will depend on the type of inputs and on the biological, physical, and chemical processes acting within the different sectors of the CWs (presence/absence of vegetation, type of vegetation, accumulation of humic substances, etc.).

## Material and methods

### Study sites

**Fig. 1:**
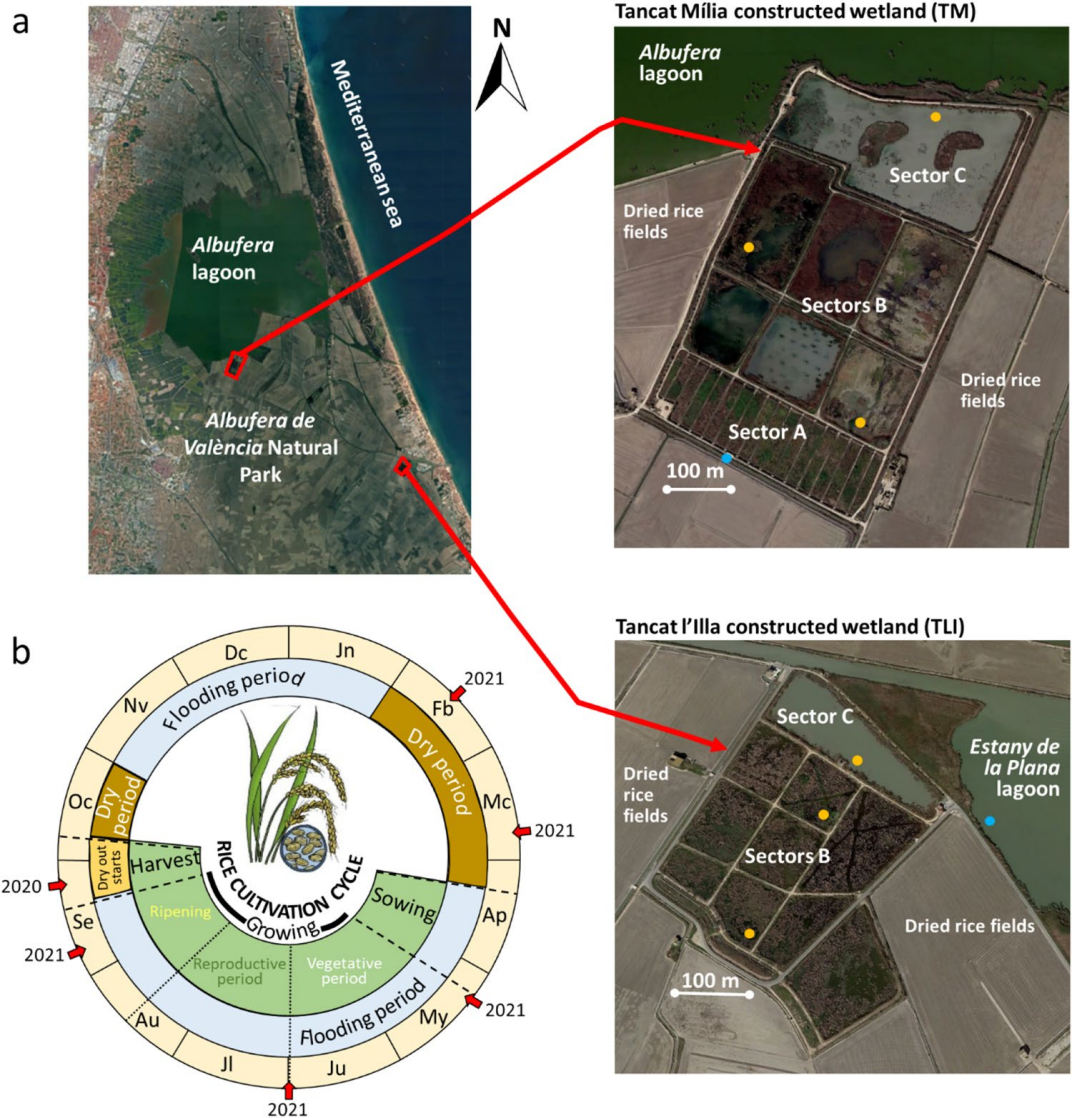
(Color online only) **a** Location of the Tancat Mília and Tancat l’Illa constructed wetlands within the *Albufera de València* Natural Park. The sampling sites within and outside each CW are indicated (orange dots are inner sites; blue dot is outer site). **b** Scheme of the hydrological cycle of the *Albufera de València* Natural Park and the one-year rice cultivation cycle. The sampling dates of the study are indicated on the one-year cycle by means of red arrows

Tancat de Mília (TM hereafter) and Tancat de l’Illa (TLI hereafter) are two CWs located to the south of the *Albufera de València* lagoon within the natural park (Fig. 1a). In this natural park, there are 21,000 ha devoted to the cultivation of rice. The TM (33.4 ha) and TLI (16 ha) CWs were formerly rice fields and were transformed into CWs in 2011. Both CWs have several sectors of surface-flow water (*B* sectors) and a shallow lagoon located at the end of the system (sector *C*). Moreover, TM has a sector of horizontal subsurface flow (sector *A*) at the beginning of the area (Fig. 1a). Since the middle of 2019, the incoming waters in both CWs have been the effluents of two sewage water treatment plants (SWTP) with tertiary treatment (*Albufera Sur* SWTP in the case of TM and *Sueca* SWTP in the case of TLI). The water flows from the inlet to sector *A* (only in TM), then pass to the *B* sectors and finally to sector C. After passing through the CWs, the water is returned to the *Albufera de València* lagoon, in the case of TM, and to a small lagoon that drains the irrigation water from the surrounding rice fields (*Estany de la Plana*), in the case of TLI. The mean water depth range is 30–60 cm in TLI, and 10–45 cm in TM. The laminar flow is 960 m^3^/day for TLI and 1400 m^3^/day for TM. Global hydraulic retention time is ca. 30 days (Hernández-Crespo et al. 2017; Martín-Monerris and Hernández-Crespo, pers. comm.).

The *B* sectors in both CWs are planted with emergent plants (mainly *Phragmites* spp., *Typha* spp., but also *Cladium mariscus*, *Scirpus* spp., *Juncus* spp., *Iris pseudacorus*; semiaquatic or amphibian plants such as *Lithrum salicaria*, *Kosteletzkya pentacarpos*, and *Hydrocotile vulgaris* are also found). In TLI, there is a predominance of *Typha* spp., and in TM *Phragmites australis* is the most abundant species followed by *Typha* spp. In sector C, submerged vegetation, composed of *Myriophyllum spicatum*, *Najas marina*, *Ceratophyllum demersum*, and *C*. *submersum* developed in TLI, forming an extensive coverage in September 2021. *Potamogeton nodosus* and *Stuckenia pectinata* had been present, but were no longer detected. However, sector C in TM remained with no submerged vegetation throughout the sampled period. For further details on the study sites see Hernández-Crespo et al. (2017) and Rodrigo and Segura (2020).

### Sample collection and analytical procedures

Three water samples were collected (at a depth of 0.2 m) within each CW, and one sample was taken outside the CWs for inside-outside comparison (Fig. 1a). The samples within the CWs were taken at the start of the system (the first part of the *B* sectors; sites I1 and M1 for TLI and TM, respectively), in the middle part of the *B* sectors (sites I2 and M2), and at the end of the system (sector *C*; site I3 and M3). The outside samples (sites “Out”) were taken in the *Estany de la Plana* lagoon in the case of TLI, and in a channel surrounding the TM CW where rice fields drain. The sampling data was mainly selected according to the representative months of the rice cultivation period (Fig. 1b) from September 2020 to September 2021, since the area is clearly affected by aerial and terrestrial pesticide treatment (Gamón et al. 2003; Gómez de Barreda Ferraz et al. 2004; Calvo et al. 2021) which occurs at the beginning of the cultivation (May), in the middle of the pesticide treatment (July) and before harvesting (September). Furthermore, samples were also taken in February and March, during the dry period. In TM, an extra sample was taken in late summer 2021 at the entrance of sector *A* (M0). This made up a total of 24 samples for TLI and 25 for TM. After collection, water samples were transported in a portable freezer to the laboratories.

The following elements were analyzed in the water within both CWs and outside: Fe, Cu, Zn, Mn, As, Pb, Cr, Ni, Cd, B, Al, and Hg. The multi-elemental analyses were performed using an Agilent 7900 ICP-MS in the support facilities for research at the University of Valencia (SCSIE).

Wide-scope screening and target quantitative analysis of pesticides were performed (Bijlsma et al. 2021). Pesticide extraction from water samples was carried out by solid-phase extraction, following the procedure described in Masiá et al. (2013). In brief, we used EB2 cartridges (Scharlab, Barcelona, Spain, 200 mg, 90 µm) preconditioned with 5 mL dichloromethane–methanol (1:1, v/v) and 10 mL deionized water. A volume of 200–400 mL sample was loaded through the cartridge using a Visiprep™ SPE vacuum manifold (Supelco, Bellefonte, PA, USA). The cartridges were dried for 10 min under vacuum and then analytes were eluted with 10 mL dichloromethane–methanol (1:1, v/v). The sample extracts were evaporated to dryness in a rotary evaporator, reconstituted with 1 mL of methanol, and filtered through 0.22 µm PTFE syringe filters.

Pesticide identification was carried out by liquid chromatography-high resolution tandem mass spectrometry (LC-HRMS/MS), using an AB SCIEX (Redwood City, CA, USA) TripleTOFTM 5600 system (at SCSIE, UV). Chromatographic separation was performed using an Acquity UPLC BEH C18 (Waters Corporation, Milford, MA, USA, 50 × 2.1 mm, 1.7 µm) column and 0.4 mL min^−1^ mobile phase of 0.1% formic acid in water and 0.1% formic acid in acetonitrile. HRMS acquisitions were carried out using electrospray ionization in positive and negative modes, using a mass range of 100–950 m/z. Data was evaluated using AB SCIEX PeakView™ software and a pesticide database of 560 compounds (see Table S1, in Supplementary Material).

### Environmental risk assessment

The environmental risk evaluation of the detected and quantified pesticides was performed based on the calculation of the risk quotients (RQs). This quotient was calculated, for each pesticide, as the ratio between the measured environmental concentration (MEC) and the predicted no-effect concentration (PNEC), the concentration at which no toxic effects are expected in aquatic organisms (Rico et al. 2013). The selection of the toxicity values in the derivation of PNECs are indicated in Table 4. The lowest PNEC values were obtained from the NORMAN Ecotoxicology Database (https://bit.ly/2Cm4zOE). The pesticides’ concentrations measured in all the within-CWs (and outside) sites during the studied period were averaged. Thus, an annual average for each CW and outside was considered for the RQ calculation. Ecological risks were determined as insignificant or negligible risk with RQ < 0.1, low risk with 0.1 < RQ ≤ 1, moderate risk with 1 < RQ ≤ 10, and high risk with RQ > 10 (García-Galán et al. 2020; Rico et al. 2013).

### Statistical analyses

We carried out the Shapiro–Wilk and the Levene tests to assess the normality of the residuals and the homoscedasticity, respectively. When both conditions were verified, ANOVAs were performed. Otherwise, non-parametric tests (i.e., Mann–Whitney and Kruskal–Wallis tests) were used for comparisons between two, or more than two data groups, respectively. In this vein, we compared the seasonal means of metal concentrations in the water of both CWs (TLI and TM). We also compared the annual means of heavy metal water concentrations between TLI and TM. The annual means of the sites outside the CWs were also compared: both with each other, and with the annual inside mean in each CW. ANOVAs or non-parametric tests were performed to compare annual means of quantified pesticides between both CWs. Statistically significant differences were considered from a probability *P* < 0.05. All the analyses were performed using the PAST 3.14 software (Hammer et al. 2001; ohammer@nhm.uio.no).

## Results

### Heavy and other metals (metalloids)

#### Tancat L’Illa (TLI)

**Fig. 2:**
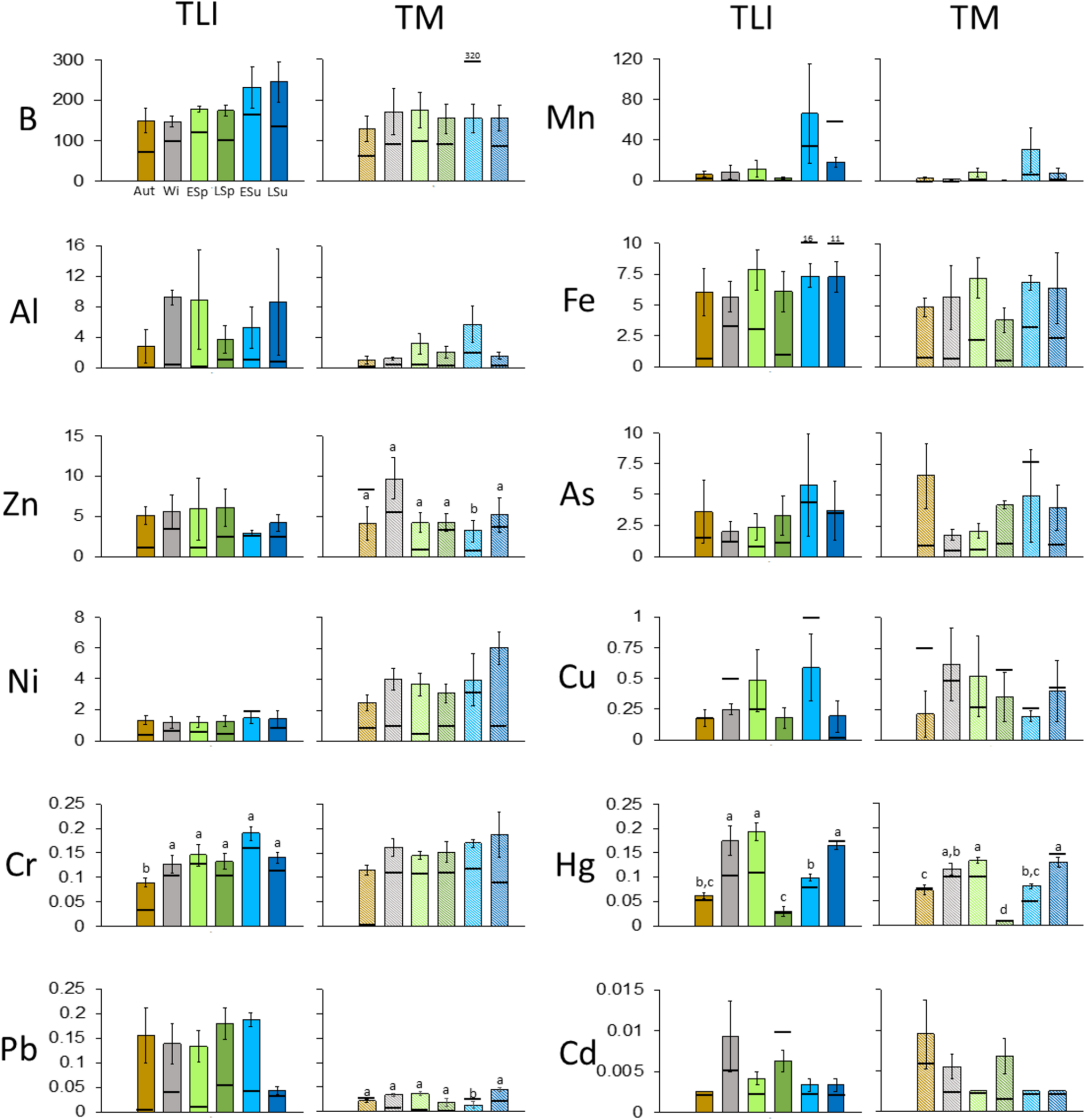
(Color online only) Seasonal means of heavy metal concentrations (all in µg L^−1^) in the water within TLI and TM (ordered by concentration). The horizontal black line on each bar indicates the concentrations outside the CWs. (Aut: autumn 2020; Wi: winter 2021; ESp: early spring 2021; LSp: late spring 2021; LSu: late summer). Thin vertical bars indicate standard error

Detailed concentrations of each element by site and by season are provided in Fig. S1 in the Supplementary Material section. The correlations among the elements are shown in Table S2. Mean Boron (B) concentrations inside the CW slightly increased from autumn–winter to late summer (although the overall differences were not statistically significant) and were always higher than in the water outside (Fig. 2). For the rest of the elements, no clear seasonal patterns were detected. The Chromium (Cr) mean concentration was statistically lower in autumn, and Mercury (Hg) was statistically lower in autumn, late spring, and early summer (Fig. 2).

Seven out of 12 (58%) of the studied elements were reduced as water passed along the several sectors of the CW (Table 1). Aluminum was the element removed with the highest mean efficiency (88% reduction), followed by Zinc (57%), and Manganese and Nickel (45%). Hg was removed by 31%, and Cr and Lead by 19% and 14%, respectively. However, B accumulated by 56%, Iron and Cadmium by approximately 30%, and Copper by more than 100%. The case of Arsenic was the most outstanding with more than 600% of mean accumulation (Table 1).

**Table 1:** Concentration removal efficiency based on differences in concentration between I1/I3 (or M1/M3) (in percentage, and expressed with a negative symbol) for several elements in both studied CWs. Accumulation percentage for other elements. Data are means of six samplings ± standard deviation, and are ordered by reduction in removal and increase in accumulation. For some elements, removal was observed in one CW and accumulation for the other CW; this is why some cells are empty and the element appears in both parts of the table

	Efficiencies (%)								
	Removal								
	Al	Cu	Zn	Mn	Ni	Hg	Fe	Cr	Pb
TLI	-88 ± 3		-57 ± 10	-45 ± 42	-46 ± 6	-31 ± 12		-19 ± 10	-14 ± 26
TM		-65 ± 13	-53 ± 12	-49 ± 22	-36 ± 8		-23 ± 17	-15 ± 12	
	Accumulation								
	Hg	Fe	Cd	B	Al	Cu	Pb	As	
TLI		29 ± 39	35 ± 29	56 ± 24		118 ± 67		644 ± 134	
TM	8 ± 9		35 ± 46	123 ± 12	192 ± 214		153 ± 151	519 ± 235	

The reduction in Zn, Ni, Hg, and Cr water concentrations caused by the CW makes their values in the outlet similar to those outside the CW (approx. 2 µg L^−1^, 1 µg L^−1^, 0.1 µg L^−1^, and 0.1 µg L^−1^, respectively; see Fig. S1). However, Al and Pb concentrations in the outlet remained higher than outside the CWs (0.76 vs. 1.51 µg L^−1^, 0.03 vs. 0.11 µg L^−1^, respectively). Mn remained lower in the outlet (16.7 vs. 4.7 µg L^−1^). Among those elements which were accumulated within the CW, the concentrations of Fe, Cd, and Cu remained quite similar in the outlet in comparison to outside the CW (6.1 vs. 6.2 µg L^−1^, 0.004 vs. 0.005 µg L^−1^, 0.4 vs. 0.5 µg L^−1^, respectively). However, B and As concentrations outside were 1.8 and 3.3 times higher than in the outlet (125 vs. 224 µg L^−1^ and 2.3 vs. 7.6 µg L^−1^, respectively).

#### Tancat Mília (TM)

No clear seasonal patterns were observed in the studied elements (Fig. 2). Only Ni and Cr showed a slight increase from autumn to summer, but with no statistical differences. Half of the studied elements were reduced as water passed through the CW (Table 1). Cu was the element which experienced the highest reduction (65%), Zn and Mn showed mean reduction efficiencies of approximately 50%, Ni and Fe 36% and 23%, respectively, and finally Cr only 15%. A slight accumulation was measured for Hg (8%), Cd accumulated by 35%. B, Al, and Pb had accumulations of more than 100%. Arsenic, as in the case of Tancat l’Illa (TLI), accumulated by more than 500% (Table 1).

The reduction of Cu and Zn caused the mean concentrations in the outlet to be lower than outside the CW (0.22 vs. 0.47 µg L^−1^ and 3.5 vs. 4.0 µg L^−1^ respectively; Fig. S2); Cr remained in similar concentrations (0.14 vs. 0.11 µg L^−1^). However, for Ni, Fe, and Mn, and despite the reduction exerted by the CW, the mean concentrations in the outlet remained higher than outside (2.9 vs. 1.2 µg L^−1^, 4.0 vs. 1.8 µg L^−1^ and 3 vs. 2 µg L^−1^, respectively). Regarding the elements accumulated within the CW, the concentrations of Cd (0.005 µg L^−1^) and Hg (0.09 vs. 0.08 µg L^−1^) remained quite similar in the outlet in comparison to outside the CW. However, B, Pb, Al, and As concentrations were 1.8, 1.9, 2.3, and 3 times higher in the outlet than outside (229 vs. 123 µg L^−1^, 0.029 vs. 0.015 µg L^−1^, 1.8 vs. 0.8 µg L^−1^ and 5.9 vs. 2.0 µg L^−1^, respectively).

Furthermore, comparing TLI and Tancat Mília (TM), it is remarkable that the pair-wise correlation among elements showed a particular pattern in each CW (Table S2).

### Pesticides

#### Tancat L’Illa (TLI)

A total of 71 pesticides (26 herbicides, 26 insecticides, and 19 fungicides) were detected in the water samples within and outside the CW (the detailed list can be found in Table S3, Supplementary Material). Out of the 71 compounds, 23 (6 fungicides, 10 herbicides, and 7 insecticides) were detected only in one sample (out of 24 samples analyzed). A total of 57 pesticides were found in the water within the CW (21 herbicides, 19 insecticides, and 17 fungicides) versus 38 pesticides outside (15 insecticides, 13 fungicides, and 10 herbicides).

**Fig. 3:**
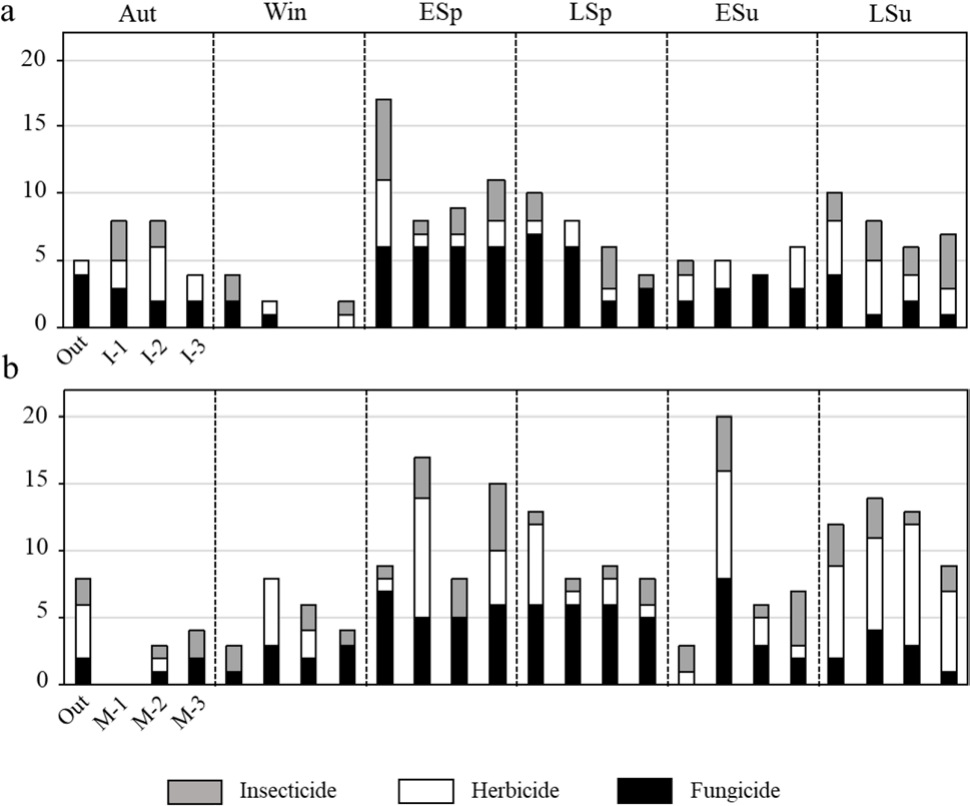
Spatial and seasonal distribution of the presence of pesticides types (herbicides, fungicides, and insecticides) within both CWs (**a**: TLI; **b**: TM) and outside. Compounds only found in one occasion have been not considered in this representation

Concerning seasonal patterns in the number of detected pesticides (Fig. 3a; see also Fig. S3; in both figures, compounds that appeared only once during the entire study period are not included), the highest number was found in early spring both inside and outside the CW (16 and 17, respectively), followed by late summer (16 inside and 10 outside). The lowest number was observed in winter (3 inside and 4 outside). Specifically, a progressive decrease in the number of fungicides and insecticides was found after early spring. The herbicides showed a gradual increase from early spring to late summer.

Regarding spatial patterns within the CW in the number of total pesticides (Fig. 3a, compounds that appeared only once during the entire study period are not included), these decreased progressively as water flowed through in autumn, late spring, and late summer. Only in early spring did the number of compounds slightly increase. By type of pesticides, the number of fungicides decreased inside the CW in autumn and late spring; the number of herbicides decreased in late spring and late summer; and the number of insecticides increased progressively inside the CW in winter, early and late spring, and late summer. In the inside-outside comparison, in most seasons, the number of pesticides outside was greater than in the water at the end of the system (I3).

The fungicides Fenpropimorph, Azoxystrobin, Carboxin, Pyrimethanil, and Tricyclazole were the most represented compounds (detected in 11, 10, and 9 out of the 24 samples; Table S3). Fenpropimorph could not be quantified but it was detected from early spring to early summer mainly within the CW. Azoxystrobin (Table 2) was detected mainly in autumn with a maximum concentration of 1 µg L^−1^ outside the CW, and a minimum of 0.009 µg L^−1^ in the I2 in late summer. Tricyclazole was found within the CW from late spring, and the maximum concentration was again outside the CW in autumn with 0.083 µg L^−1^. Carboxin was found both within and outside the CW only in the spring samplings. Pyrimethanil was found in most seasons and sites (with the exception of autumn and late summer). Tebuconazole and Thiabendazole were less represented, and the maximum concentration was 0.1 µg L^−1^ for the former and 3.7 µg L^−1^ for the latter (Table 2).

**Table 2:** Concentration (all in µg L.^−1^) of some pesticides detected within both CWs and outside in the different seasons (Aut: autumn 2020; Wi: winter 2021; ESp: early spring 2021; LSp: late spring 2021; LSu: late summer). M0*: an extra water sample taken before the subsurface section in TM CW only in late summer (“nd” indicates there is no data)

Family	Compounds	Season	Sites in TLI				Sites in TM				
			Out	I1	I2	I3	Out	M0*	M1	M2	M3
Fungicides											
*Methoxyacylates*	Azoxystrobin	Aut	1.027	0.445	0.549	0.089	0.530	nd		0.117	0.103
*(Strobilutins)*		ESp	0.033	0.041	0.022		0.105	nd	0.103	0.140	0.095
		LSp					0.025	nd	0.039	0.014	
		ESu	0.079					nd			
		LSu	0.471		0.009		0.349	0.035	0.036	0.018	0.034
*Anilide*	*Meta*laxyl	ESp		0.039				nd			
*Conazole*	Tebuconazole	Aut		0.105				nd			0.078
		Wi		0.024				nd			
		ESp		0.042		0.025	0.006	nd			0.018
		LSp	0.010				0.009	nd	0.047		0.025
		ESu						nd	0.045	0.009	
		LSu	0.032								
*Benzimidazole*	Thiabendazole	ESp		0.619				nd			
		LSp	0.523	3.758				nd			
		ESu						nd	1.674		
		LSu						1.463	1.333		
*Benzothiazole*	Tricyclazole	Aut	0.083					nd			
		Wi	0.010					nd			0.006
		ESp						nd			0.009
		LSp	0.010	0.018		0.012		nd		0.013	0.010
		ESu	0.025		0.013	0.017		nd	0.008		0.013
		LSu				0.013					
Herbicides											
*Phenylurea*	Diuron^1^	Aut			0.110			nd			
		ESp						nd	0.008		
		LSp						nd	0.012	0.021	
		ESu				0.010		nd	0.066		
Insecticides		LSu		0.005						0.003	
*Carbamate*	Carbaryl	LSu						0.225			
*Carbamate*	Carbofuran	Aut		0.447	0.353			nd		0.231	0.244
		ESu						nd	0.166		
		LSu	0.060			0.065		0.060	0.077	0.036	0.047
*Organophosphate*	Dimethoate	ESu						nd	0.684		
*Neonicotinoid*	Imidacloprid^2^	ESu						nd	0.083		

^1^Compound included in the List of Priority substances in the field of water policy (Directive 2013/39/EU). The annual mean in inland surface waters listed Real Decreto 817/2015 for diuron is 0.2 µg L^−1^ and the maximum allowable concentration is 1.8 μg L^−1^

^2^Compound included in the Watch List (Commission implementing decision (EU) 2018/840)

The herbicides Clethodim and Hexazinone were the most represented compounds (Table S3), found in 9 and 6 samples (out of 24). Clethodim-imin-sulfone was detected from early spring to late summer, but its spatial representation decreased with time. Hexazinone was found mainly within the CW and spatially scattered. Diuron was detected on three occasions with maximum concentrations of 0.11 µg L^−1^ in I2 (Table 2).

The insecticides Carbofuran and Jasmolin I and the synergist Piperonyl butoxide were the most represented compounds (Table S3), each found on 4 occasions. Carbofuran showed the highest concentrations in autumn in I1 and I2 (approximately 0.4 µg L^−1^; Table 2). Jasmolin I was found only within the final sectors of the CW in spring and late summer. Piperonyl butoxide was detected only in the cold seasons (Table S3).

#### Tancat Mília (TM)

A total of 71 pesticides (29 herbicides, 23 insecticides and 19 fungicides) were detected within and outside TM (the detailed list can be found in Table S3, Supplementary Material). Out of the 71 compounds, 21 (5 fungicides, 6 herbicides and 10 insecticides) were detected only in one sample (out of 25 samples analyzed). A total of 65 pesticides (28 herbicides, 19 insecticides, and 18 fungicides) were found in water samples within the CW, versus 29 pesticides outside (10 herbicides, 10 insecticides, and 9 fungicides).

With regard to seasonal patterns (Fig. 3b and Fig. S3), the maximum number of pesticides within the CW was reached in early spring (25 compounds), with a large decline immediately after, and the minimum number in autumn (4 compounds). In contrast, outside the CW, the maximum was observed in late spring (12 compounds) and the minimum in winter and early summer (3 compounds). Both inside and outside the CW, the number of fungicides experienced a progressive increase until spring, followed by a decrease later on. Only inside the CW did the herbicides and insecticides increase their presence from autumn to early spring.

Regarding spatial patterns (Fig. 3b), a decrease in the total number of pesticides was observed as the water flowed through the CW in winter, early spring, and early and late summer. With regard to the types of pesticides, the number of fungicides decreased in late spring and early and late summer; the number of herbicides decreased in winter, early spring, and early summer, and the number of insecticides decreased only in late summer. In the inside-outside comparison, only in half of the seasons was the water (M3) returned outside with a lower number of pesticides than the number found in the surroundings.

The fungicides Azoxystrobin, Pyrimethanil, Fenpropimorph, Carboxin, and Tebuconazole were the most represented compounds (detected in 15, 12, 11, 9, and 8 out of the 25 samples). Azoxystrobin was found in all seasons and sites, with the exception of early summer (Table 2; Table S3). The maximum concentration was 0.53 µg L^−1^ outside the CW and the minimum 0.018 µg L^−1^ in M2 in late summer (Table 2). Pyrimethanil was present from winter, with higher concentrations in M1 (see Table S3). Fenpropimorph was detected from early spring, both within and outside the CW, and its relative concentration decreased later on. Carboxin was detected only during the spring in all sites. Tebuconazole was detected from early spring until early summer, with maximum concentrations of 0.047 µg L^−1^ in M3 in late spring. Tricyclazole was found only within the CW, mainly at the end of the system (M3), with maximum concentrations of 0.013 µg L^−1^. Thiabendazole was found only in three samples in summer, with maximum concentrations of 1.7 µg L^−1^ (Table 2). None of the quantified fungicides showed significant differences in the annual means between both CWs (Table S4).

The herbicides Clethodim, Sebuthylazine, Simazine, Terbuthylazine, Hexazinone, and Diuron were the most represented compounds (Table S3), found in 8, 7, 6, and 5 samples (out of 25). Clethodim-imin-sulfone was detected from early spring to late summer mainly inside the CW and with higher relative concentrations than in TLI (see Table S3). Sebuthylazine-desethyl, Simazine, and Terbuthylazine-desethyl were found outside the CW from autumn, but with higher relative concentrations within the CW in late summer. Hexazinone was found only within the CW with higher relative concentration in early spring. Diuron was also detected only inside the CW, mainly in sector B, with a maximum concentration of 0.07 µg L^−1^ in I2 (Table 2). As occurred with fungicides, the mean annual concentration of the quantified Diuron was not significantly different between TLI and TM (Table S4).

The insecticides Carbofuran Jasmolin I, Tebufenozide, and Piperonyl butoxide were the most represented compounds (Table S3), each found on (7) 8 occasions. Carbofuran was found exclusively within the CW mainly in late summer; the highest concentrations were measured in autumn in M2 and M3 (approximately 0.2 µg L^−1^; Table 2). Jasmolin I was found only in early spring within all sectors of the CW as well as outside, but its relative concentration decreased as water passed along the CW. Piperonyl butoxide was detected mainly in early summer, but the highest relative concentration was found in autumn outside the CW (Table S3). Tebufenozide was represented from late spring, and the higher relative concentrations at M1 were substantially reduced in M2 and M3. Dimethoate and Imidacloprid were detected sporadically (1 occasion each) with concentrations of 0.7 µg L^−1^ and 0.08 µg L^−1^, respectively (Table 2). Again, there were no significant differences in the mean annual concentrations of insecticides between both CWs (Table S4).

### Risk assessment of the quantified pesticides

Regarding the environmental risk assessment for freshwater (Table 4), the calculated RQs show that most pesticides pose negligible or low risk both within and outside the CWs. The fungicide Thiabendazole and the insecticide Imidacloprid are the only ones posing a moderate risk for both CWs in the first case, and only for TM, in the second.

## Discussion

### Heavy metals and other trace elements

Tertiary treatment is of importance when treated wastewater is discharged into sensitive ecosystems such as wetlands, rivers, lakes or used in groundwater regeneration or agriculture. Tertiary treatment in wastewater treatment plants may use chemical reagents (coagulants, flocculants) to increase nutrient removal efficiencies (Iborra-Clar et al. 2011). TLI may have received wastewater enriched in Al, Zn, Ni, Hg, Pb, and Cr, since the concentrations of these elements are much higher in the first sector (which receives the wastewater directly without passing through any sub-superficial-flow sector) of the CW than in the receptor waterbody (*Estany de la Plana*). *Sueca* sewage water treatment plant uses aluminum polychloride as a coagulant in the tertiary treatment (the most widely used coagulant type at present; Rahmadyanti et al. 2021), as well as a cationic polyelectrolyte in previous treatments. However, the water concentrations of Al, and the rest of the elements, are efficiently reduced as water flows through the different sectors of the CW. Several studies have reported the reduction of heavy metals through microbial and plant biomass in CWs, by means of two main mechanisms: the active one being energy-dependent bioaccumulation, and the passive one energy-independent biosorption (by the adsorption mechanism) (Malyan et al. 2021; Yu et al. 2021). For example, Bianchi et al. (2021) reported biosorption through macrophyte species, such as *Phragmites australis* (the role of reed biomass as a biosorbent material yields the removal of Zn by 73%, under optimum dose and experimental conditions). Previously, Southichak et al. (2006) also reported the biosorption of Ni, Pb, and Zn through reed biomass. The above-cited mechanisms (facilitated by the presence of emergent vegetation, mainly *Typha* spp., but also some *Phragmites* spp., and the microbial activity in the water and sediment; Wang et al. 2022) explain the reduction of these metals in TLI. It is worth pointing out that this water removal effect, particularly for Al, is totally produced within the first *B* sector, since water from last *B* sector and *C* sector already have concentrations equal to the surrounding waters. The wide water surface and slow flow of the aerobic zone of wetlands enhances the bacterial metal oxidation and the following hydroxylation, which causes the precipitation of Fe, Mn, and Al hydroxides (Luko-Sulato et al. 2021). Moreover, the precipitated oxyhydroxides of Fe and Mn can absorb other heavy metals, such as Pb, Ni, and Cr (Matagi et al. 1998). Mn is also oxidized by the microorganisms from the bivalent Mn (II) to tetravalent Mn (IV) and then Mn (IV) is precipitated as manganese oxide (Malyan et al. 2021). Moreover, in wetlands, the complexation of metals can be facilitated by the presence of organic molecules (e.g., tannic, fulvic, and humic acids) which are nucleophile or mostly multidentate ligands that interact with heavy metals, influencing their solubility and mobility (Weng et al. 2002). The presence of such organic molecules is evident in TLI, particularly in I2 (the last *B* sector) whose water has an evident reddish color.

For the other elements, TLI functions the opposite way; B, Fe, Cd, Cu, and As are accumulated in the CW waters. Boron is a metalloid which is widely used in several industrial activities, such as the production of cleaning products, glass, and agrochemicals. Due to its high solubility, it is easily found in anthropic waters (Türker et al. 2014). Atmospheric precipitation and aerosols are another input of B to aquatic systems, along with marine salts. Thus, in water bodies close to the sea, as is the case of the TLI, relatively high concentrations are expected. However, the use of perborate products increases B concentrations in domestic wastewater, and little or no B is removed during conventional wastewater treatment processes (Türker et al. 2014). The results of domestic wastewater discharge can contribute to the elevated B concentration inside the CW compared to the site outside (Fig. 2). In the *Albufera* lagoon, the B concentrations are also lower (130 ± 13 µg L^−1^ as annual mean for 2020–2021; CHJ 2022). The chemistry of B differs from that of other trace elements, and the overall B removal process in CWs is very complex, making the identification of specific removal pathways more difficult (Türker et al. 2014). Several experiments in microcosms and mesocosms have documented B removal, but the rates of removal in full-scale CWs is low (see references in the review of Türker et al. 2014). Several environmental factors such as pH, temperature, B speciation, and seasonal effects can affect the B concentration in CWs water. The highest accumulation within TLI is observed in early and late summer, and this can be explained because both the solubility rates of B compounds and the dissociation of boric acid increase as temperature increases (Tu et al. 2010). Copper compounds are commonly used in agriculture to treat plant pests or for water treatment (Zandi et al. 2020). The different use in agriculture depending on the season would explain the different concentrations of Cu outside the TLI (maximum of 1 µg L^−1^ in early summer and undetectable values in late summer). The mean Cu accumulation rates in TLI are mainly due to the warm period data; the rest of the year no accumulation happens, but nor was a reduction observed, as has been described for other CWs treating domestic wastewater (Arroyo et al. 2010). Cd can proceed from the manufacture and application of phosphate fertilizers. The performance of TLI on Cd, although yielding an annual mean accumulation as water passed along the CW, when the system received the highest concentrations (as high as 0.018 µg L^−1^ in winter time), can reduce it to almost undetectable values at the outlet. Arsenic has a predominantly geogenic or mining-derived origin in Iberian Peninsula paddy field soils (Signes-Pastor et al. 2016) and deserves special attention due to its accumulation in TLI, since it has been described how the stable complexation of As, in association with dissolved organic matter and Fe and solid phase humic substances (Mandal et al. 2019), act as the main sinks of As in water-saturated sediment, as in the case of these CWs. But, at the same time, the increase in As in outlet concentrations has been amply reported in the literature for different types of CWs (Arroyo et al. 2010 and references therein). Arsenic has the highest concentrations in sector C in TLI, a pond-like waterbody with emergent vegetation only on the shores and with moderate coverage of submerged vegetation only during the warmer periods. It seems that the highest As concentrations in the pond happen during the moments of greater coverage of submerged plants (mainly *Myriophyllum spicatum* and also *Najas marina* which outcompete *M*. *spicatum* in the late summer). One could expect that the submerged plants could have changed the redox potential of the sediment to more oxidized conditions and produced the sink of As. However, it seems that the conditions developed in sector *C* by submerged vegetation were not able to maintain stable oxidized conditions to guarantee As removal by combination with oxides (Pedescoll et al. 2015). On the other hand, the higher presence of humic acids in the *B* sectors in comparison to sector C could be responsible for the lower As concentrations, due to the complexation capacity on metalloids, such as As, of these substances (Mandal et al. 2019). More attention should be paid to humic substance composition in CWs, particularly facing climate change since, as Lipczynska-Kochany (2018) pointed out, this has a clear induced impact on humic compounds and their interactions with soil and water pollutants such as metals. Regarding Hg, one of the most toxic heavy metals, particularly in its methylated form (Regnell and Watras 2019), TLI is capable of “neutralizing” the higher concentrations probably arriving with the wastewater in winter and early spring.

**Fig. 4:**
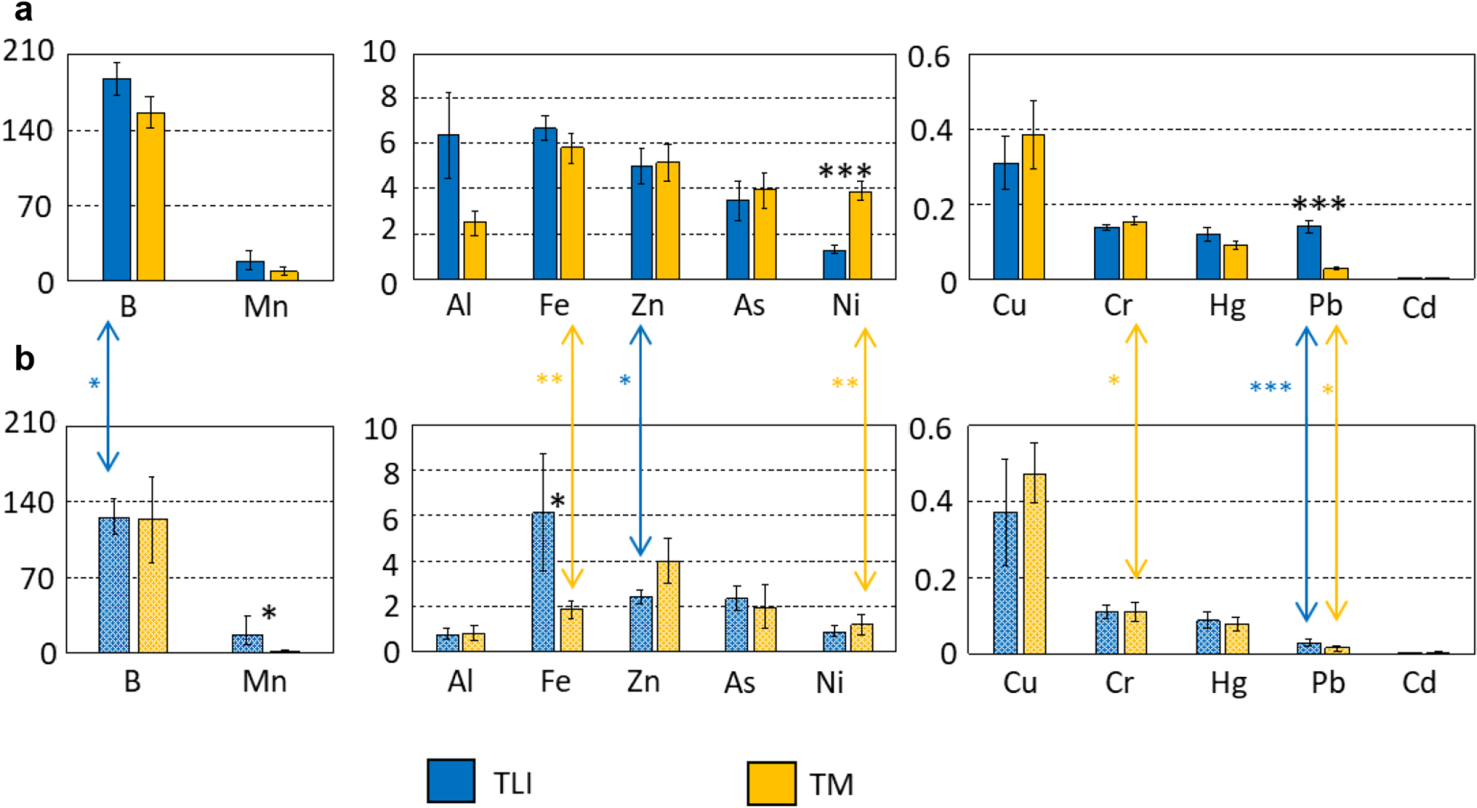
(Color online only) Comparison of annual means of metal concentrations (all in µg L^−1^) in the waters (sites grouped) within TLI and TM (**a**) and for sites outside the CWs (**b**). Statistically significant differences among element means between TLI and TM and between inside and outside the CWs (the arrows) are indicated by asterisks (*p < 0.05; **p < 0.01; ***p < 0.001)

The performance of TM is very similar to that of TLI for several elements (very similar removal for Zn, Mn, Ni, and Cr; similar accumulation for Cd, B, and As) and the mechanisms involved in both CWs must be the same since the design of the two CWs is similar. Regarding Cd, this CW, again, is capable of reducing the highest concentrations in autumn in the inlet to less than a half in the outlet. However, a relevant difference is the concentrations of Al between the two CWs, and the explanation is because, in the case of TM, aluminium polychloride is not used as a coagulant in the tertiary treatment in the *Albufera Sur* sewage water treatment plant. Instead, ferric chloride is used, and this must be the reason why Fe has a statistically higher concentration inside the CW rather than outside (Fig. 4). A source of Pb in the area can be related to the use of lead ammunition, since the Natural Park had been used for hunting activities practiced for decades until the implementation of Real Decreto 581/2001 (which was not totally effective until 2003), which banned the use of Pb ammunition for hunting in internationally relevant wetlands (Valverde et al. 2019). Lead shot pellets remained in the sediments for years and the construction of the CWs, using machinery, could have moved deeper sediments corresponding to the hunting period upwards. This would explain why the annual Pb means are significantly higher within the CW compared to outside both CWs (Fig. 4). The statistically higher Ni mean concentrations within TM in comparison to TLI and outside (and in the *Albufera* lagoon: < 1.2 µg L^−1^; CHJ 2022) are related to the input water, since in five out of the six occasions the Ni concentration was clearly higher in M1 (see Fig. S4). On the other hand, one important difference between both CWs is the presence of a sub-superficial-water flow sector (*A*) only in TM. However, it seems its existence is not relevant for the concentrations of most of the elements, since there were no statistically significant differences between the mean annual concentrations when I1 and M1 were compared. Only B, Al, and Pb show a 30%, 82%, and 84%, respectively, lower values in TM than in TLI (Fig. S4). Aluminum differences have been discussed above.

### Metal concentrations to be discharged from both CWs in comparison to legal restrictions

TLI manages to reduce (Mn) or to maintain at the same level the concentration of most of the studied elements (Zn, Ni, Hg, Cr, Fe Cd, Cu) as outside (67%). Only Al, Pb, B, and As are at higher concentrations. TM also reduces Zn and Cu, and maintains the concentration of Cr, Cd, and Hg (which represents 42%). Al, Pb, B, and As remain at higher concentrations, as occurs in TLI, but also Ni, Fe, and Mn are at higher concentrations. The European Union establishes the environmental quality standards for priority substances in continental waters, registered for Spain in *Real Decreto 817*/*2015* (*BOE 219*, *12*–*09*-*2015*). The annual means of Pb concentrations in the outlets of TLI and TM (0.11 ± 0.06—standard deviation—and 0.03 ± 0.02 µg L^−1^, respectively) are far lower than the EU quality standards (7.2 µg L^−1^). These means for B in the outlets of TLI (224 ± 78 µg L^−1^) and TM (229 ± 42 µg L^−1^) are also below the limit recommended by the World Health Organization (500 µg L^−1^). For Al (1.5 ± 0.5 µg L^−1^ and 1.8 ± 1.5 µg L^−1^ for TLI and TM, respectively), which is not listed in the standards cited above, the limit for drinking water is 200 µg L^−1^. Even for As (7.6 ± 3.7 µg L^−1^ and 5.9 ± 4.4 µg L^−1^), which is the element with a higher concentration in the outlet in comparison to outside the CWs, both CW mean concentrations are far below the quality standards for priority substances in continental waters (50 µg L^−1^) or other superficial waters (25 µg L^−1^). Ni concentrations in TM (2.9 ± 1.1 µg L^−1^) are one order of magnitude lower than the EU standards (20 µg L^−1^). Fe and Mn are not listed in the EU standards cited above, but the limit for drinking water is established at 200–300 µg L^−1^ and 50 µg L^−1^ respectively, and in the TM outlet both mean concentrations (4 ± 2 µg L^−1^ and 3 ± 3 µg L^−1^, respectively) are clearly below these thresholds.

For the rest of the elements, mean concentrations in both CW outlets are also below the limits of the quality standards of the EU for priority substances in continental waters (hexavalent Cr = 5 µg L^−1^, Cr = 50 µg L^−1^; Cu = 5–120 µg L^−1^, and Zn = 30–500 µg L^−1^ depending on the water hardness; Cd < 0.08 µg L^−1^ and ≤ 0.45 µg L^−1^ as admissible maximum). Hg is the only element whose mean concentrations in both outlets (0.10 ± 0.06 µg L^−1^ and 0.09 ± 0.05 µg L^−1^ for TLI and TM, respectively) are above the limit for priority substances (0.05 µg L^−1^), and even slightly higher than the admissible maximum concentration (0.07 µg L^−1^). Overall, the waters surrounding the CWs also have annual mean concentrations slightly above these limits (0.09 and 0.08 µg L^−1^ for sites outside TLI and TM, respectively), since these areas receive waters discharged from the rice fields with agrochemicals and pesticide residues derived from the rice farming activity, as occurs in other areas of the Spanish Mediterranean coast (Salvadó et al. 2006). In the *Albufera* lagoon, no Hg values above 0.02 µg L^−1^ were measured in our study period (CHJ 2022). However, in previous years, one-time values of 0.08–0.09 µg L^−1^ were recorded in October 2018 and January 2019.

### Pesticides

The presence of pesticides in both CWs is related to the fact that they are immersed in an agrolandscape mainly based in the cultivation of rice, but also other crops (citrus, vegetables, potatoes, etc.; Gamón et al. 2003; Köck et al. 2010; Hedge et al. 2014; Rico et al. 2018; Calvo et al. 2021). Several compounds are used as treatments to control pests annually by means of aerial and terrestrial methods in the 14.000 ha covered by rice fields within the Natural Park. Although both CWS showed the same elevated number of pesticides in their water, the composition was not exactly equal. Moreover, 32% (TLI area) and 29% (TM area) of the compounds were detected only in a single sample, and 19% (TLI) and 13% (TM) in only two samples (Table 3). The number of pesticides which are present in more than 25% of samples is low, and of the same order as those compounds found in more than 40% of samples (3%). TM is clearly more affected by pesticides than TLI, not only regarding the number of pesticides (35 versus 24 exclusive compounds in TM and TLI, respectively) temporally but also spatially (see Table S3). The application of pesticides in the Natural Park is somehow different depending on the particular area. Although there are some general rules/legislation (Generalitat Valenciana 2008, 2020), each area is treated slightly differently. The rice cultivation treatments start with the use of herbicides against the weeds, and only when rice plants are growing are the fungicides used to avoid or reduce the presence of plagues responsible for rice diseases (Calvo et al. 2021). In our case, the number of herbicides detected in early spring was one of the highest, particularly for TM. The herbicides Clethodim-imin-sulfone (a metabolite of Clethodim) and Hexazinone were found within both CWs, but mainly in TM. Clethodim is a graminicide applied postemergence to control annual grasses (Jordan 2011) and it is usually applied combined with other graminicides (Jordan et al. 2003). Diuron, found also within both CWs (more sporadically in TLI), classified as priority substances in Annex X Directive, 2000/60/EEC, did not surpass the annual means (0.2 µg L^−1^) or the maximum concentrations (1.8 µg L^−1^) established in Annex II of Directive, 2013/39/EU for continental surface waters (or Real Decreto 817/2015). The herbicide Bentazone, one of the two most abundant pesticides in the *Albufera de València* Natural Park aquatic systems in 2016 (Calvo et al. 2021), although detected in the surroundings of TM in March 2021, it was not found within TM or TLI. The application of this herbicide by tractor in the rice fields prevented its presence within both CWs. The three herbicides Simazine, Terbuthylazine-desethyl, and Terbutryn were found exclusively in TM and their pattern of appearance is very similar (mainly in the last sampling: late summer). Simazine is also referred in Annex II of Directive, 2013/39/EU for continental surface waters (or Real Decreto 817/2015), but we cannot say if it surpasses, or not, the annual means (1 µg L^−1^) or the maximum concentrations (4 µg L^−1^) since we were not able to quantify it, but probably not, since Simazine has been also detected in *Albufera de València* lagoon waters (southern part) with mean concentrations for 2020–2021 of 0.015 ± 0.007 µg L^−1^ (CHJ 2022). The use of Terbutryn was forbidden by Regulation (EC) 2076/2002; we could not quantify it, but in the south part of the *Albufera* lagoon it is < 0.01 µg L^−1^ (CHJ 2022). Terbuthylazine-desethyl is a residue of Terbuthylazine, which remains approved. These compounds are non-specific herbicides which have been widely used in the production of corn, wheat, and potatoes, among others (Chhokar and Sharma 2008; Stipičević et al. 2015; Hormenoo et al. 2021).

**Table 3:** Number of pesticides separated by type present exclusively outside the CWs, exclusively within the CWs, in common in the different areas. The number of compounds that appear only in one or two samples, or between 25 and 40% of samples or in more than 40% of samples is also indicated (total samples for TLI: 24; for TM: 25). The percentage is referred to 71 pesticide compounds found in each CW area

	Out			Within CW			In common Out-Within		Only 1 sample (4%)		Only 2 sample (8%)		> 25%, but < 40% samples		> than 40% samples	
Pesticide	excl. TLI	excl. TM	In common	excl. TLI	excl. TM	In common	TLI	TM	TLI area	TM area	TLI area	TM area	TLI area	TM area	TLI area	TM area
Herbicides	7	7	3	10	16	11	6	8	6	5	5	1	1	1	0	0
Fungicides	4	2	7	6	7	11	10	8	10	6	4	7	2	3	2	2
Insecticides	11	5	4	8	12	10	8	5	7	10	5	1	0	1	0	0
**Total**																
**(number)**	**22**	**14**	**14**	**24**	**35**	**32**	**24**	**21**	**23**	**21**	**14**	**9**	**3**	**5**	**2**	**2**
***Total (%)***									***32***	***30***	***20***	***13***	***4***	***7***	***3***	***3***

Fungicides were the most represented pesticides, and 12 were common to both CWs (Table 3). Azoxystrobin and Difenoconazole are applied together under the brand name of Amistar Top (Syngenta, Basilea, Switzerland) and are supplied in two runs, at the end of July and at the end of August, on the rice fields by helicopter (*Generalitat* Valenciana, 2021); the former was detected both outside and within the CWs (42% and 60% of samples in TLI and TM, respectively), and the highest concentrations were found in late September 2020 and early September 2021. Its presence within the CWs is evidently related to the application method, since the CWs are completely surrounded by rice fields. However, Difenoconazole was detected sporadically, mainly outside the CWs and with a higher presence at the end of September 2020. This compound degrades more easily and rapidly than Azoxystrobin (Meenakshi et al. 2007). The fungicide Tricyclazole, found within both CWs, is a post-emergence fungicide which was usually sprayed from mid-July until the rice harvest; however, this active substance was not approved by Commission Implementing Regulation (EU) 2016/1826 and, thus, all existing authorizations for plant protection products containing Tricyclazole were revoked. Nevertheless, the use of this chemical has been allowed in some years as a terrestrial and aerial treatment by the Agricultural Ministry of Spain with exceptional authorizations (it could be sprayed twice in one treatment after an interval of 15–20 days). In 2016, Calvo et al. (2021) found that Tricyclazole was dominant (88–100%) from the beginning of rice cultivation in the four aquatic systems analyzed (the *Albufera* lagoon, rice fields, irrigation, and outlet channels), with peak values in September 2016 (3.3 to 6.6 µg L^−1^ in channels and rice fields and 5.9 µg L^−1^ in the *Albufera* lagoon). Currently, our values are much lower (maximum of 0.08 µg L^−1^ at the end of September 2020). Tricyclazole has been described by its high solubility, its persistence in the water and the lack of stability to photolysis in the water. However, based on results of an experimental field study, Tsochatzis et al. (2013) reported that Tricyclazole is not really persistent in rice field water, probably due to degradation or volatilization processes and its adsorption by the field sediments. Our results were able to corroborate this observation since its concentrations in the water were low. It also could indicate that this fungicide is currently applied (although it does not appear in the list of permitted substances; MAPA, 2020) in lower doses. We have no information about how and when this fungicide was applied in 2020–2021. Two more fungicides were found in both CWs, Tebuconazole and Thiabendazole. Tebuconazole has been one of the most common and abundantly found in the *Albufera de València* Natural Park in other studies (Calvo et al. 2021), also currently in the southern part of *Albufera* lagoon at concentrations < 0.01 µg L^−1^ (CHJ 2022). Tebuconazole is used post-harvest not only in rice but also in citrus crops. This agrochemical is used at the beginning of July and could be used until August or September depending on the strength of the pyricularia (*Magnaporthe oryzae*) disease in the year of the cultivation. It has low or moderate solubility and is bio-accumulative; it has been reported how its persistence in water field samples does not last more than 4–5 days (Fu et al. 2016). This is why the concentrations found in our study are not high (maximum of 0.1 µg L^−1^). Thiabendazole is not used for rice cultivation, but it is used in other crops such as citrus (oranges and tangerines) and potatoes which are also cultivated within the area of the Natural Park. Calvo et al. (2021) found this compound in concentrations lower than 0.1 µg L^−1^, but we detected it in higher concentrations, occasionally at 3.7 µg L^−1^. The fungicide Carbendazim, which was prohibited before 2016, was detected just in one sample within TLI. Merel et al. (2018) reported that textiles and papers commonly found in households could be a source of Carbendazim in domestic wastewater. Thus, the effluents of the wastewater treatment plants might be a source of this compound. Some other fungicides which were applied in the past in the area, such as Propiconazole, and that are currently forbidden, were not detected.

The insecticides Carbofuran and Jasmolin I, and the synergist Piperonyl butoxide, were common in both CWs, and they were the most represented; along with Tebufenozide in TM. Carbofuran, Jasmolin I, and Tebufenozide were not detected by Calvo et al. (2021) in 2016. Carbofuran is a systemic granular insecticide frequently used to combat insects in rice cultivation (Das et al. 2003). In Spain, it was excluded from the list of permitted phytosanitary products (MAPA 2020) following ONU guidelines where this compound is included in the Consolidated List of Prohibited or Restricted Products. Jasmolin I is an insecticide from the group of pyrethrines mainly affecting the nervous system of diverse insects. Furthermore, Piperonyl butoxide is commonly used as an insecticide synergist which enhances the active properties of pyrethrines (Schleier III et al. 2008). This fact could explain the joint presence of these compounds in both CWs. Tebufenozide is a synthetic insect growth regulator which exhibits insecticidal activity by inducing premature and incomplete molting of larvae of various lepidopteran insect pests (Gómez de Barreda Ferraz et al. 2004). These authors assessed the effect of this insecticide on phytoplanktonic species from the *Albufera de València* Natural Park. The neonicotinoid insecticides Acetamiprid and Imidacloprid are usually applied in the area at the beginning of July by tractor. Both insecticides were the most detected ones in the July water samples in the study by Calvo et al. (2021). The dissipation of neonicotinoids seems to be largely dominated by photolysis and temperature (Rico et al. 2018). Both are soluble in water and have a low capability of bioaccumulation; Acetamiprid persist in the water less than a week. All this, together with the type of application, are the reasons why they were not detected in our samples (only Imidacloprid was found within TM in M1 in early summer with < 0.1 µg L^−1^).

One of our aims was to assess how pesticide composition can be modified as water provided by the wastewater treatment plants passes along the CWs. We have seen that the most ubiquitous pesticides (e.g., Azoxystrobin and Difenoconazole) arrive by aerial dispersal. But the water course throughout the different sectors of the CWs causes the decrease in the prevalence of most pesticides. Several processes occurring within the CWs, such as adsorption, hydrolysis, microbial degradation, sedimentation, photolysis, and plant uptake play an important role in said removal of pesticides in CWs (Vymazal and Březinová 2015; Malyan et al. 2021). Moreover, the concentrations of pesticides that have been quantitatively analyzed (Table 2) are far below the toxic concentrations for aquatic organisms that have been reported in several studies. For fungicides, Rodrigues et al. (2013) carried out a review on Azoxystrobin in aquatic systems and, in the wide variety of organisms studied the toxicity of this compound was above 2 µg L^−1^, with some organisms being able to tolerate concentrations several orders of magnitude higher. Regarding Metalaxyl, studies with *Daphnia magna* (Chen and Liu 2008) and cyanobacteria (Hamed et al. 2020) showed a lethal concentration 50 (LC_50_) of more than 50 mg L^−1^ and 25 mg L^−1^, respectively. It has been reported, in laboratory studies, that Tebuconazole applied in concentrations from 500 µg L^−1^ has negative effects on microbial communities (Zubrod et al. 2011). Martín-de-Lucía et al. (2019) demonstrated in a microcosm experiment that Thiabendazole had toxic effects on *D*. *magna* at a concentration in the water from 9 mg L^−1^. For Tricyclazole, Rossaro and Cortesi (2013) reported an LC_50_ from 20 mg L^−1^ for different aquatic species from microfauna to fish (e.g., *Cyprinus carpio*). As for herbicides, for Diuron, an LC_50_ has been reported ranging from 4.3 to 42.0 mg L^−1^ in fish, and from 1.0 to 2.5 mg L^−1^ in aquatic invertebrates (Giacomazzi and Cochet 2004). Regarding insecticides, Gunasekara et al. (2008) analyzed different aquatic species, both invertebrates and vertebrates, at different stages (larvae, juveniles, and adults), establishing the toxicity of Carbaryl in a range between 3.6 µg L^−1^ and 34.7 mg L^−1^, depending on the species. With respect to Carbofuran, Dobšíková (2003) compared and analyzed the toxicity of this compound in different aquatic and terrestrial species, establishing a range of 0.04–13.08 mg L^−1^ as the LC_50_. For Dimethoate, several studies have analyzed its toxicity in aquatic organisms such as fish (toxic effects at concentrations above 1.84 mg L^−1^; Singh et al. 2009) or rotifers (toxic effects at concentrations above 0.18 mg L^−1^; Guo et al. 2012). Tišler et al. (2009), in an experimental setup, showed that Imidacloprid was not as toxic to some aquatic organisms as other environmental pollutants, mainly affecting *D*. *magna* individuals at concentrations above 1.3 mg L^−1^. When the environmental risk of quantified pesticides was assessed (RQ for freshwater), most compounds showed negligible to low risks (Table 4). The higher environmental risk obtained for Imidacloprid in TM could be attributed to their very low PNEC value as it was concluded also by García-Galán et al. (2020). Nevertheless, more in-depth study must be undertaken in this vein, with a quantification of more pesticides that could be present throughout the year and in more sites both within and outside the CWs, and with a better assessment of PNECs, due to the large disparity of reported values for the freshwater aquatic environment (see Table 4).

**Table 4:** Environmental risk of pesticides detected and quantified in Tancat l’Illa (TLI), Tancat de Mília (TM) and outside the CWs (Out) as risk quotient (RQ) calculated as the proportion between the measured environmental concentration (MEC) and the predicted no-effect concentration (PNEC) for each pesticide. The MEC values were calculated as an annual average for each CW (and outside). The lowest PNECs for freshwater (PNEC_lowest_, in italics) were obtained from the NORMAN Ecotoxicology Database (https://bit.ly/2Cm4zOE). The reference for the PNEC value used for QR calculation for each pesticide is provided. Negligible risk (< 0.1); low risk (0.1–1); moderate risk (1–10); high risk (> 10) (García-Galán et al. 2020)

Pesticides	TLI			TM		Out		*Reference
	*PNEC*_*lowest*_-PNEC* (µg/l)	MEC (µg/l)	RQ	MEC (µg/l)	RQ	MEC (µg/l)	RQ	
Fungicides								
Azoxystrobin	*0.2*–1	0.19	0.19–Low	0.07	0.07–Negligible	0.33	0.33–Low	Rodrigues et al. 2017
Metalaxyl	*9.47*–20	0.04	0.00–Negligible					Carere et al. 2021
Tebuconazole	*0.24*–1	0.05	0.05–Negligible	0.04	0.04–Negligible	0.01	0.01–Negligible	Stamatis et al. 2010
Thiabendazole	*3.3*–1.2	2.19	1.82–Moderate	1.49	1.24–Moderate	0.52	0.44–Low	Pérez-Villanueva et al. 2022
Tricyclazole	*0.47*–1.8	0.01	0.01–Negligible	0.01	0.01–Negligible	0.03	0.02–Low	Xie et al. 2020
Herbicides								
Diuron	*0.07*–0.2	0.04	0.21–Low	0.02	0.11–Low			García-Galán et al. 2020
Insecticides								
Carbaryl	*0.23*–0.54			0.23	0.42–Low			Vryzas et al. 2011
Carbofuran	*0.016*– 0.8	0.29	0.36–Low	0.12	0.15–Low	0.06	0.08–Negligible	Vryzas et al. 2011
Dimethoate	*0.07*–4			0.68	0.17–Low			Vryzas et al. 2011
Imidacloprid	*0.013*–0.01			0.08	8.30–Moderate			García-Galán et al. 2020

## Final remarks and conclusions

Overall, Zn, Mn, Ni, and Cr water concentrations were reduced, whereas Cd, B, and As water content increased as water passed along both CWs. TLI is capable of dampening the above-legal-limit concentrations of Hg supplied by wastewater at certain times, and reducing them to levels closer to the European Union’s legal limits for environmental quality. Although both CWs vary with regard to metal removal, no risks to human health or the environment have been detected due to the low metal concentrations in the outlets. However, the use of synthetic coagulants is disadvantageous due to the residual aluminum content they produce, since although the CWs are capable of removing them from the water, they can accumulate in the sediments and/or the biota and can be deleterious in the medium/long-term. Therefore, natural organic coagulants should be seen as an attractive alternative to chemicals because of their abundant availability, low cost, non-toxicity, and multifunctionality (Tukki et al. 2016; Rahmadyanti et al. 2021). The way the pesticides are applied in the rice fields and surrounding crops of the CWs (tractor, bag spraying, helicopter) plays a determining role in their presence in the CWs waters. With the wide-scope screening methodology applied in this study, we have seen that TM is much more affected by pesticides than TLI. However, the mechanisms facilitated by the spatial structure of the CWs, (e.g., plant uptake by the continuous presence of emergent and submerged—occasionally—macrophytes, adsorption, microbial degradation, sedimentation, hydrolysis, photolysis, etc.) must play an important role in pesticide removal in the CWs. Now, the next step is to quantify by target quantitative analyses the presence of the remaining most frequently used pesticides to unravel whether or not they are above the legal limits and to calculate refined risk assessments. Moreover, their presence in the sediment of the CWs and the biota (Braschi et al. 2022) needs further analyses in future campaigns, as well as the participation of microorganisms in key processes of pollutant degradation (Wang et al. 2022).

Finally, we want to stress the crucial environmental services that both CWs provide to this particular agrolandscape immersed in a protected area of international importance for biodiversity, in terms of reducing the environmental impacts of treated domestic wastewater, pesticide application in the surroundings and improving water quality and safety.

## Supplementary Information

Below is the link to the electronic supplementary material.

Supplementary file1 (DOCX 1656 KB)

## Data Availability

Not applicable.
