# Supplementary material for: Two constructed wetlands within a Mediterranean natural park immersed in an agrolandscape reduce most heavy metal water concentrations and dampen the majority of pesticide presence
Source: Environ Sci Pollut Res Int. 2022 Jun 17;29(52):79478–96. doi: 10.1007/s11356-022-21365-w (PMC9587099; doi:10.1007/s11356-022-21365-w)
Supplement: Supplementary file 1 — Supplementary file1 (DOCX 1656 KB) [file 11356_2022_21365_MOESM1_ESM.docx]

**SUPPLEMENTARY MATERIAL for “Two constructed wetlands within a Mediterranean natural park immersed in an agrolandscape reduce most heavy metal water concentrations and dampen the majority of pesticide presence”** by Rodrigo et al.

**Fig. S1** Heavy and other metals (metalloids) concentrations (all in µg L^-1^) in the water within TLI and outside in several seasons (I1: inlet; I2: intermediate site; I3: outlet; Out: outside the CW). The green circle in the graphs for early summer corresponds to a sample taken directly in inlet of the treated wastewater. The elements are ordered according their concentrations (from higher to lower concentrations)

 **Fig S1** Continuation

**Fig. S2** Heavy and other metals (metalloids) concentrations (all in µg L^-1^) in the water within TM and outside in several seasons (M1: inlet; M2: intermediate site; M3: outlet; Out: outside the CW). The elements are ordered according their concentrations (from higher to lower concentrations).

**Fig. S2** Continuation.

**Fig. S3** Seasonal distribution of the number of pesticides types (herbicides, fungicides and insecticides) within both CWs (above-left: TLI; above-right: TM) and outside (below). Compounds only found in one occasion have been not considered in this representation

**
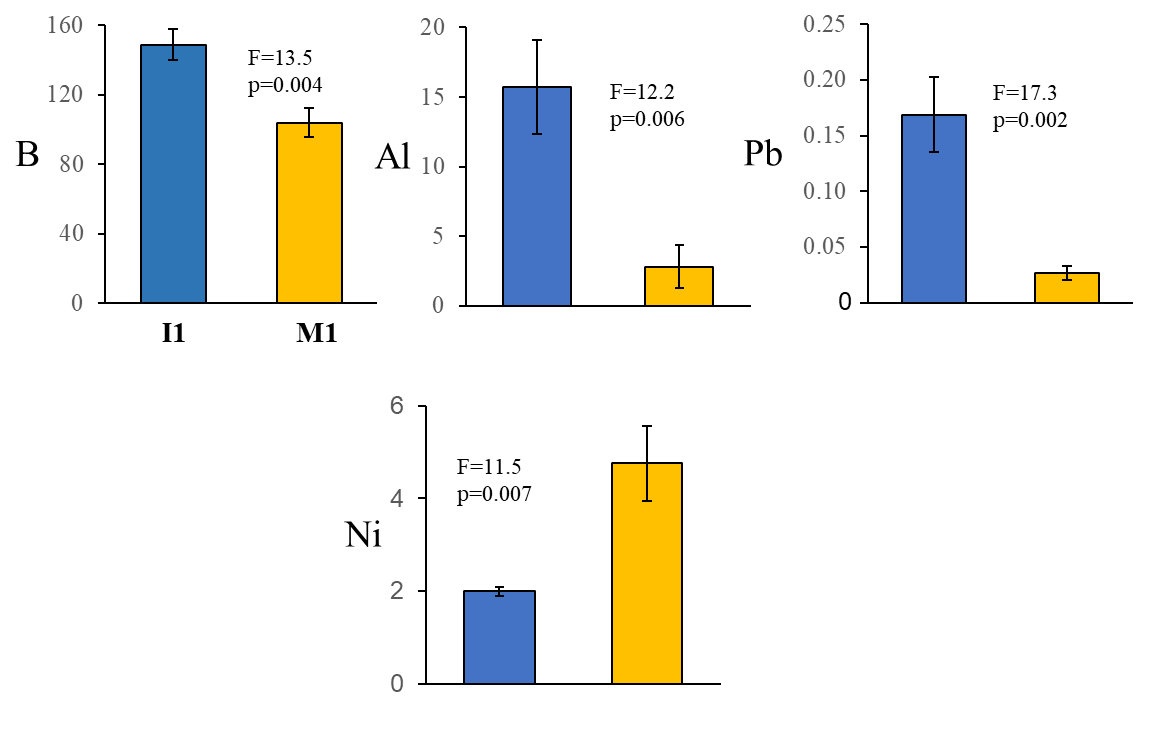
**

**Fig. S4** Mean annual B, Al, Pb and Ni concentrations (all in µg L^-1^) in I1 and M1. The thin bars indicate the standard error (n=6). This are the only element which concentrations were statistically different (ANOVA F and probability is indicated)

**Table S1** List of compounds against the screened signals were compared

**Table S2** Significant Pearson correlation coefficients between elements in each sampling site for each CW (above) and considering all sampling sites together (below). *p<0.05; **p<0.01;***p<0,001 (negative correlations are indicated in red)


**Table S3** List (ordered alphabetically) of pesticides (fungicides, herbicides and insecticides) found in the wide-scope screening performed on the water samples within both CWs (I1-I3; M1-M3) and outside (Out) in the different seasons (Aut: Autumn 2020; Wi: Winter 2021; ESp: early Spring 2021; LSp: late Spring 2021; LSu: late Summer). M0*: an extra water sample taken before the subsurface-flow section (sector *A*) in TM only in late summer. A colour code is used for relative presence (comparing all sites analysed) of each compound. For those compounds analysed quantitatively, the numbers indicate concentration in µg L^-1^

**Table S4** Results of the statistical analyses to compare the mean values of the pesticides quantitatively (shown in Table 2) analysed on both CWs areas
